# CD146 as a Prognostic-Related Biomarker in ccRCC Correlating With Immune Infiltrates

**DOI:** 10.3389/fonc.2021.744107

**Published:** 2021-12-08

**Authors:** Zheng Lv, Hua-Yi Feng, Wang Tao, Hong-Zhao Li, Xu Zhang

**Affiliations:** ^1^ School of Medicine, Nankai University, Tianjin, China; ^2^ Department of Urology, The Third Medical Center, Chinese People Liberation Army (PLA) General Hospital, Beijing, China; ^3^ Medical School of Chinese People Liberation Army (PLA), Beijing, China

**Keywords:** CD146, ccRCC, prognosis, methylation, tumor microenvironment

## Abstract

**Backgrounds:**

CD146 is highly expressed in various malignant tumors and associated with the poor prognosis. However, the role of CD146 in clear cell renal cell carcinoma (ccRCC) is still unknown. This study aimed to identify the role of CD146 in ccRCC by integrated bioinformatics analysis.

**Methods:**

CD146 mRNA expression and methylation data in ccRCC was examined using the TIMER, UALCAN, and MethSurv databases. CD146 expression in paraffin-embedded tissues (140 cancer samples and 140 paracancer tissues) from our cohort were examined by immunohistochemistry assay. The LinkedOmics database was used to study the signaling pathways related to CD146 expression. TIMER and TISIDB were used to analyze the correlations among CD146, CD146-coexpressed genes, tumor-infiltrating immune cells, and immunomodulators. The relationship between CD146 and drug response in renal cancer cell lines was analyzed by the CTRP and CCLE databases.

**Results:**

The mRNA and protein levels of CD146 were elevated in ccRCC tissues than that in paracancer tissues. The DNA methylation of CD146 in ccRCC tissues were lower than that in normal tissues. Importantly, high CD146 expression was associated with poor prognosis in patients with ccRCC. Furthermore, multivariate Cox regression analysis showed that CD146 was an independent prognostic factor in ccRCC. GO and KEGG pathway analyses indicated the co-expressed genes of CD146 were mainly related to a variety of immune-related pathways, including Th1 and Th2 cell differentiation, Th17 cell differentiation, and leukocyte transendothelial migration. Our data demonstrated that the expression and methylation status of CD146 were strongly correlated with immune infiltration levels, immunomodulators, and chemokines. Further, the sensitivity and resistance of renal cancer cell lines to some drugs were related to CD146 expression.

**Conclusions:**

Our study highlights the clinical significance of CD146 in ccRCC and provides novel insights into the immune function of CD146 in the tumor microenvironment.

## Introduction

Clear renal cell carcinoma (ccRCC) is the most common subtype of renal cell carcinoma (1). Despite substantial advancement in ccRCC target therapies, such as tyrosine kinases inhibitors and mTOR inhibitors, the prognosis for advanced and metastatic ccRCC patients remains poor (2, 3). ccRCC is a highly immune-infiltrated tumor (4). In patients with metastatic RCC, immunotherapy-based combinations have become the standard of care and show an efficacy and overall survival benefit in the first-line metastatic setting (5, 6). The interaction between tumor cells and the tumor microenvironment (TME) provides new insights into the molecular drivers underlying ccRCC occurrence, metastasis, and recurrence (7, 8). However, the molecular mechanisms underlying ccRCC carcinogenesis remain unclear.

CD146, also known as MUC18, is a highly glycosylated type I transmembrane protein. Normal expression of CD146 is restricted to certain cell types, including endothelial cells (9), fibroblasts (10), smooth muscle cells (11), and lymphocytes (12). CD146 is weakly expressed or not detected in normal adult tissues but is strongly upregulated under various pathological conditions such as atherosclerosis (13), inflammation (14), and tumorigenesis (15). Accumulating evidence confirmed that CD146 was highly expressed on advanced primary and metastatic cancers including gastric cancer (16), melanoma (17), and lung cancer (18). The overexpression of CD146 could promote tumor progression and metastasis by altering the expression of genes in cancer cell proliferation, apoptosis, and angiogenesis (19, 20). While the expression of CD146 and clinical significance in ccRCC is still unknown.

Growing evidence suggested that CD146 could promote the tissue-infiltrative potential and augment inflammatory response in several inflammatory diseases, including systemic sclerosis (21), rheumatoid arthritis (22), and inflammatory bowel disease (23). CD146 participates in the regulation of local immunity by recruiting mononuclear cells from the peripheral blood to the site of inflammation (24). CD146 also induces the formation of cytoplasmic protrusions and acts as an endothelial adhesion receptor, thereby mediating lymphocyte adhesion, transmigration, and lymphocyte homing (25). Previous studies have shown that inflammation affects the progression of cancer, as the chronic inflammation persists, the risk of carcinogenesis increases (26). Netti GS et al. reported that the expression of PTX3 can affect immunoflogosis in the ccRCC microenvironment, by activating the classical pathway of CS (C1q) and releasing pro-angiogenic factors (C3a, C5a), thus playing an effect on resident cells to sustain carcinogenesis (27). Detection of these markers can provide information on early diagnosis, treatment effect, and prognosis of related malignant tumors (28, 29). Krishna Y et al. show that M2 type macrophages dominate in metastatic uveal melanoma and contribute to an immunosuppressive TME by upregulation of CD146 (30). Nevertheless, the role of CD146 in affecting the components in TME in ccRCC is still poorly understood.

Here, we present a comprehensive analysis of CD146 in ccRCC using multiple available databases. We found that CD146 is significantly overexpressed in ccRCC, and CD146 expression is associated with tumor stage, tumor grade, and prognosis in ccRCC patients. The co-expressed genes of CD146 were enriched in pathways involved in endothelium development, response to virus, T cell activation, and adaptive immune response. Both CD146 expression and its methylation status were correlated with tumor infiltrating immune cells and immunomodulators in ccRCC. More importantly, we also explored the potential of using CD146 as a possible therapeutic target in ccRCC treatment. Our study indicated that CD146 may be used as a prognostic biomarker and new immune-associated therapeutic target for ccRCC patients.

## Materials and Methods

### Tumor Immune Estimation Resource Analysis

The Tumor Immune Estimation Resource (TIMER) (https://cistrome.shinyapps.io/timer/) web server is a resource that systematically analyzes immune infiltrates across different cancer types (31). To evaluate the expression difference of CD146 between tumor and adjacent normal tissues, we used the TIMER database to study the RNA sequence data of different cancer types in TCGA (The Cancer Genome Atlas). The immune cell abundance was estimated by the TIMER algorithm. Correlation modules were used to determine the relationship between the RNA-seq expression profile data of CD146 in ccRCC and immune cells, including CD4+ T cells, CD8+ T cells, regulatory T (Treg) cells, T follicular helper (Tfh) cells, Type 1 T helper (Th1) cells, Type 2 T helper (Th2) cells, natural killer (NK) cells, myeloid dendritic cells, monocyte, neutrophils, M1 macrophages, and M2 macrophages. The gene markers of immune cells were also correlated with CD146 expression using gene modules. These gene markers referenced are cited in previous publications (32–34).

### DNA Methylation Analysis

DNA methylation during carcinogenesis has an impact on not only gene expression, but also the prognosis of cancer patients (35). MethSurv (https://biit.cs.ut.ee/methsurv/) is a web portal that provides survival analysis based on DNA methylation biomarkers using TCGA data. DNA methylation of CD146 at CpG sites and the prognostic value of these CpG sites in ccRCC were analyzed by MethSurv.

### Patients and Clinical Materials

Tumor specimens were collected from 140 ccRCC patients diagnosed with ccRCC treated with radical or partial nephrectomy at the Department of Urology of the Chinese People’s Liberation Army (PLA) General Hospital (Beijing, China) from January 2013 to December 2019. The medical records of clinic-pathologic data from our institutional database, including age, gender, T stage, N stage, M stage, and Fuhrman grade, were retrospectively reviewed. All patients were staged according to the eighth edition of the AJCC-UICC TNM classification (36). Fuhrman classification was used to attribute nuclear grade (37). The clinic-pathologic data are reported in **Table 1**. All samples of cancer tissue had been pathologically confirmed as ccRCC by two pathologists. All patients were informed and signed a consent for the use of clinical data for scientific purposes. The present study was approved by the ethics committee of the Chinese PLA General Hospital.

**Table 1:** Relationship between CD146 expression and clinicopathological features in patients with ccRCC.

Variable	No. of patients (%)			*χ^2^*	*p*-value
	Patients	CD146 high	CD146 low		
Age (years)					
≤60	104	50(48.1)	54(51.9)	1.126	0.289
> 60	36	21(58.3)	15(41.7)		
Gender					
Male	108	53(49.1)	55(50.9)	0.509	0.476
Female	32	18(56.3)	14(43.8)		
T stage					
T1+T2	106	49(46.2)	57(53.8)	3.517	0.061
T3+T4	34	22(64.7)	12(35.3)		
N stage					
N0	128	64(50.0)	64(50.0)	0.305	0.581
N1	12	7(58.3)	5(41.7)		
M stage					
M0	134	66(49.3)	68(50.7)	2.668	0.102
M1	6	5(83.3)	1(16.7)		
Fuhrman grade					
Grade 1 + 2	100	39(59.6)	61(40.4)	19.215	0.000
Grade 3 + 4	40	32(83.7)	8(16.3)		

### Immunohistochemistry

To determine CD146 protein expression in ccRCC, IHC staining of CD146 was conducted on cancer and paracancer tissues for 140 ccRCC cases from our cohort. For IHC staining, a tissue microarray (TMA) was obtained from the tissue bank at the Department of Urology of the Chinese PLA General Hospital. IHC staining of TMA tissues was performed with antibodies against CD146 (Abcam, ab75769). The standard protocols were followed as previously described (38). Slides were scanned using an Axio Image Z2 Microscope (Zeiss) and the TissueFAXS imaging system (TissueGnostics GmbH, Austria). All images were analyzed by TissueQuest and StrataQuest software (TissueGnostics GmbH, Austria). Staining intensity was scored 0 (negative), 1 (weak), 2 (moderate), and 3 (strong). Staining range was scored on a 4-point scale (0 = 0%, 1 = 1%∼24%, 2 = 25%∼49%, and 3 = 50%∼100%). The final IHC score was obtained by multiplying the intensity scores with the staining range. ccRCC patients with a final IHC score ≥4 were included in the high CD146 group, whereas those with a final IHC score<4 were included in the low CD146 group (39).

### Western Blot

Western blot assays were used according to standard techniques as previously reported (40). Antibodies against CD146 (ab75769; Abcam) and β-actin (#3700; CST) were used.

### UALCAN Analysis

UALCAN (http://ualcan.path.uab.edu/) is a web resource for online analysis of gene transcriptional data and clinical data of cancers from TCGA (41). We obtained differential expression of CD146 mRNA and protein of CD146 in ccRCC tumor tissues and adjacent normal tissues in the UALCAN database. DNA hypermethylation at promoters can lead to gene silencing (42). To further identify the mechanisms underlying the upregulation of CD146 in ccRCC, the methylation levels of CD146 in the ccRCC dataset were also analyzed by the UALCAN database. The differential expression of CD146 and its promoter methylation status in patients with various tumor grade (grades 1, 2, 3, and 4), tumor stage (stages 1, 2, 3, and 4) and ccRCC subtype (ccA and ccB) were also compared.

### LinkedOmics Database Analysis

The LinkedOmics database (http://www.linkedomics.org/login.php) is acknowledged as a web portal that analyses multi-omics data from TCGA datasets (43). We searched for the differentially expressed genes related to CD146 in ccRCC using the LinkFinder module. The correlation results were analyzed by the Pearson correlation coefficient and were visualized by volcano plot and heat maps. To obtain description information, the differentially expressed genes related to CD146 were annotated using Gene Ontology (GO) analysis, Kyoto Encyclopedia of Genes and Genomes (KEGG) pathway enrichment analysis, and gene set enrichment analysis (GSEA) *via* the LinkInterpreter module.

### TISIDB Database Analysis

The TISIDB database (http://cis.hku.hk/TISIDB) is a web server for the interplay between the tumor and immune system, which can assist in the prediction of immunotherapy responses (44). In our study, the associations between CD146 expression and lymphocytes, immunomodulators, and chemokines were analyzed by the TISIDB database. A ‘rho’ value greater than 0.2 and less than −0.2 was considered as a significant correlation at p < 0.05 (45).

### Correlation Between CD146 and Drug Response

To explore whether CD146 could be used as a therapeutic target in cancer patients, we investigated the correlation between CD146 expression and drug response. CD146 gene expression levels in 30 cancer cell lines were obtained from the Cancer Cell Line Encyclopedia (CCLE; https://portals.broadinstitute.org/ccle/). Drug response data in cancer cell lines were downloaded from the Cancer Therapy Response Portal (CTRP, https://portals.broadinstitute.org/ctrp.v2.2/) (46). The correlation between CD146 expression and drug response area under the curve (AUC) was analyzed by Pearson correlation coefficient analysis in each cancer cell type. The percentage of drugs significantly correlated with CD146 was obtained. The ratio of drugs related to CD146 in 10 different cancer types with 30 cell lines was represented by histogram. The correlation between CD146 and 545 drug responses under the curve in 21 renal cancer cell lines were analyzed by SangerBox and shown by volcanic plots. We considered a p-value of less than 0.05 as statistically significant.

### Statistical Analysis

Statistical analysis was performed using the GraphPad Prism version 8.0 (GraphPad software, USA) and Statistical Package for Social Sciences (SPSS 17.0 for Windows, SPSS, Chicago, IL). The measurement data are presented as mean ± SD. An independent samples t test was used to analyze the differential expression levels of CD146 mRNA between the ccRCC tissues and the adjacent normal tissues from TCGA databases. Correlations between CD146 expression and clinicopathological characteristics were analyzed by the Pearson’s Chi squared test. Overall survival (OS) analysis and progression-free survival (PFS) analysis were performed by Kaplan–Meier plots and the differences were compared using the log-rank test. Univariate and multivariable analyses were performed using the Cox proportional hazards regression models. A two-tailed P value of 0.05 was considered statistically significant.

## Results

### CD146 Is Upregulated in ccRCC

**Figure 1:**
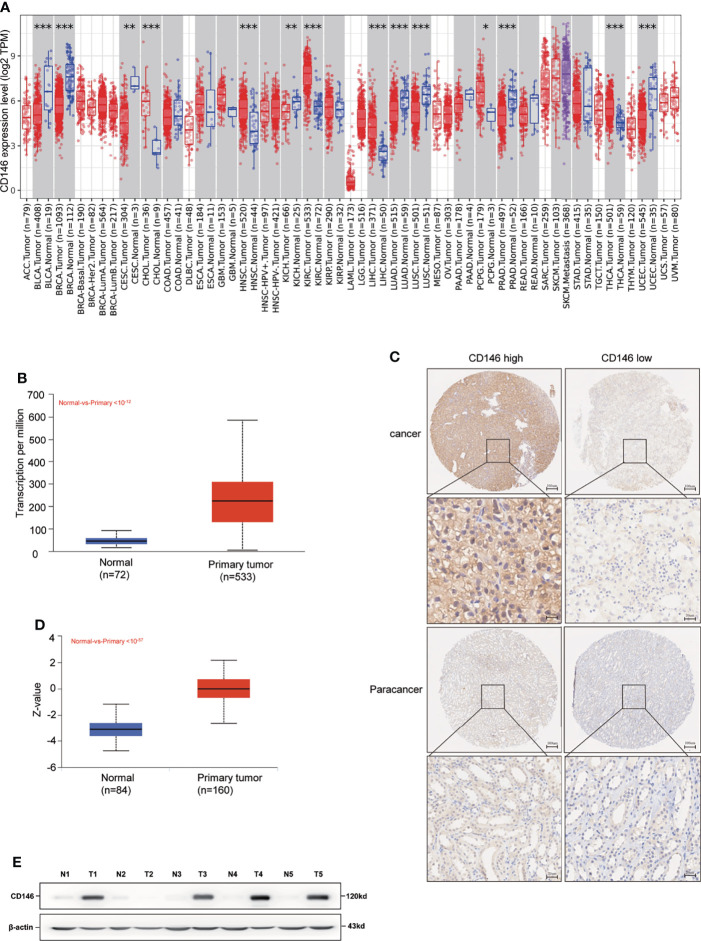
High Expression of CD146 in ccRCC. **(A)** Human expression levels of CD146 in various malignant tumor types from The Cancer Genome Atlas (TCGA) database were analyzed by the Tumor Immune Estimation Resource (TIMER). CD146 was upregulated in cholangiocarcinoma (CHOL), head and neck squamous cell carcinoma (HNSC), kidney renal clear cell carcinoma (KIRC), liver hepatocellular carcinoma(LIHC), pheochromocytoma and paraganglioma (PCPG), prostate adenocarcinoma (PRAD), and thyroid carcinoma (THCA) cancers, and downregulated in bladder urothelial carcinoma (BLCA), breast invasive carcinoma (BRCA), cervical squamous cell carcinoma and endocervical adenocarcinoma (CESC), kidney chromophobe (KICH), lung adenocarcinoma (LUAD), lung squamous cell carcinoma (LUSC), and uterine corpus endometrial carcinoma (UCEC) cancers. *p < 0.05. **p < 0.01. ***p < 0.001. **(B)** Gene Expression Profiling Interaction Analysis (UALCAN) for the expression of CD146 mRNA in tumor tissues and normal tissues based on TCGA samples. **(C)** Representative immunohistochemistry images of CD146 in ccRCC cancer tissues and corresponding normal tissues. **(D)** Protein level of CD146 in normal tissues and ccRCC cancer tissues using CPTAC samples by the UALCAN database. **(E)** Protein expressions of CD146 in five pairs of ccRCC and adjacent normal tissues samples were determined by western blot assay (N: normal tissues, T: ccRCC cancer tissues).

Compared with normal tissues, CD146 was upregulated in cholangiocarcinoma, head and neck squamous cell carcinoma, kidney renal clear cell carcinoma, liver hepatocellular carcinoma, pheochromocytoma and paraganglioma, prostate adenocarcinoma, and thyroid carcinoma cancers, while CD146 was downregulated in bladder urothelial carcinoma, breast invasive carcinoma, cervical squamous cell carcinoma and endocervical adenocarcinoma, kidney chromophobe, lung adenocarcinoma, lung squamous cell carcinoma, and uterine corpus endometrial carcinoma cancers (**Figures 1A, B**). Consistent with the mRNA expression data, we found that CD146 protein was highly expressed in the ccRCC cancer tissues compared with paracancer tissues (**Figure 1C**). The positive staining of CD146 was mainly located in the plasma and membrane (**Figure 1C**). The results of UALCAN database analysis further confirmed that CD146 protein expression was higher in primary ccRCC cancer tissues than that in paracancer tissues (**Figure 1D**). The western blot assay also confirmed that CD146 is higher in ccRCC tumor tissues than that in paracancer tissues (**Figure 1E**). This evidence strongly indicated that the mRNA and protein expressions of CD146 were significantly upregulated in ccRCC.

### The Prognostic Value of CD146 and Its Correlation With Clinicopathological Parameters in ccRCC

**Figure 2:**
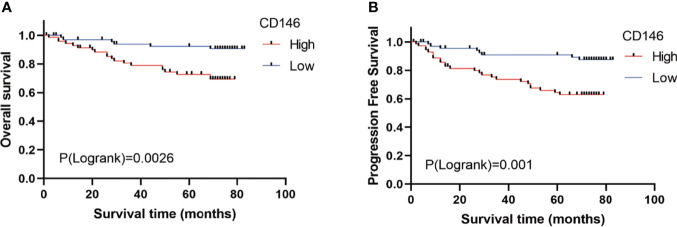
The prognostic value of CD146 in patients with ccRCC. **(A, B)** Kaplan–Meier survival analysis revealed that ccRCC patients with high CD146 expression exhibited a shorter overall survival **(A)** and progression-free survival **(B)** than that in patients with low CD146 expression.

As is shown in **Table 1**, CD146 was more expressed in higher grade cancers compared to lower grades cancers (**Table 1**). Kaplan-Meier analysis demonstrated that high expression of CD146 was significantly associated with poor OS [hazard ratio (HR) = 3.677, p = 0.0028] and PFS (HR = 3.493, p = 0.0009) in ccRCC patients (**Figures 2A, B**). Univariate Cox regression analyses showed an association between OS with age, T stage, N stage, M stage, Fuhrman grade, and CD146 expression. Moreover, multivariate Cox regression analysis showed that CD146 expression (HR = 4.655, p = 0.007), Fuhrman grade (HR = 3.472, p = 0.005), and M stage (HR = 3.625, p = 0.004) were independent prognostic factors for ccRCC patients (**Table 2**). Univariate Cox regression analysis indicated that T stage, N stage, M stage, Fuhrman grade, and CD146 expression were correlated with PFS. Furthermore, multivariate Cox regression revealed that CD146 expression (HR = 5.829, p = 0.001), Fuhrman grade (HR = 2.927, p = 0.007), and M stage (HR = 3.028, p = 0.005) were independent prognostic indicators for ccRCC patients (**Table 3**). These results suggest that CD146 was upregulated in ccRCC and associated with worse prognosis.

**Table 2:** Univariate and multivariable Cox regression of CD146 expression for overall survival in ccRCC patients.

Variable	Univariate Cox regression			Multivariable Cox regression		
	HR	95%CI	*p*-value	HR	95%CI	*p*-value
Age: >60 vs ≤60	0.431	0.212∼0.874	0.020	0.739	0.335∼1.632	0.455
Gender: male vs female	0.967	0.416∼2.244	0.967			
T stage: T3+T4 vs T1+T2	3.668	1.761∼7.638	0.001	1.252	0.502∼3.123	0.630
N stage: N1 vs N0	4.416	1.680∼11.609	0.003	3.136	0.859∼11.444	0.084
M stage: M1 vs M0	8.994	4.146∼19.510	0.000	3.625	1.495∼8.789	0.004
Fuhrman grade: G3+G4 vs G1+G2	7.456	3.423∼16.245	0.000	3.472	1.455∼8.284	0.005
CD146: high vs low	8.117	2.833∼23.255	0.000	4.655	1.525∼14.207	0.007

**Table 3:** Univariate and multivariable Cox regression of CD146 expression for progression-free survival in ccRCC patients.

Variable	Univariate Cox regression			Multivariable Cox regression		
	HR	95%CI	*p*-value	HR	95%CI	*p*-value
Age: >60 vs ≤60	0.730	0.366∼1.453	0.370			
Gender: male vs female	1.227	0.539∼2.793	0.626			
T stage: T3+T4 vs T1+T2	3.816	1.967∼7.400	0.000	1.360	0.605∼3.060	0.457
N stage: N1 vs N0	4.255	1.759∼10.295	0.001	2.397	0.809∼7.104	0.115
M stage: M1 vs M0	7.891	3.841∼16.213	0.000	3.028	1.393∼6.581	0.005
Fuhrman grade: G3+G4 vs G1+G2	6.302	3.140∼12.648	0.000	2.927	1.341∼6.390	0.007
CD146: high vs low	8.897	3.405∼23.245	0.000	5.829	2.110∼16.103	0.001

### DNA Methylation of CD146 and Its Prognostic Value in ccRCC

**Figure 3:**
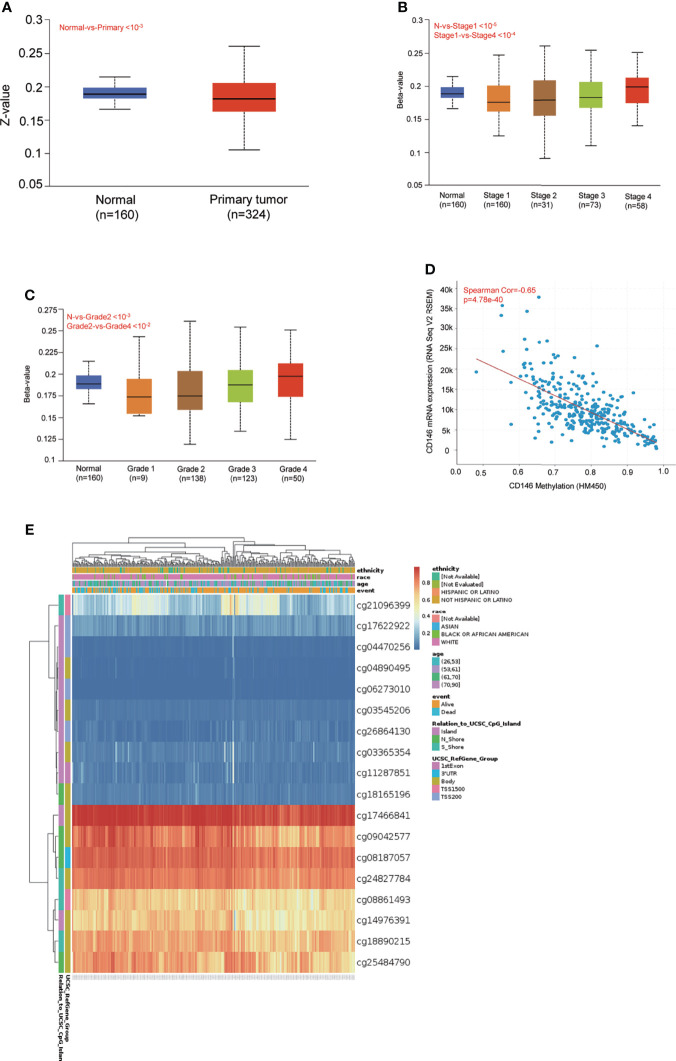
DNA methylation levels of CD146 and its prognostic value in ccRCC. **(A)** Promoter methylation level of CD146 in normal tissues and primary ccRCC tissues by the UALCAN database. **(B, C)** Promoter methylation level of CD146 in ccRCC cancer tissues of various tumor stage **(B)** and tumor grade **(C)** by the UALCAN database. **(D)** Correlation analysis of CD146 mRNA expression with CD146 promoter methylation status by the UALCAN database. **(E)** The heat map of DNA methylation at CpG sites in the CD146 gene by the MethSurv database.

DNA methylation levels of CD146 were significantly lower in ccRCC cancer tissues compared with normal samples (**Figure 3A**). The methylation status of CD146 was high in late-stage and high-grade tumors (**Figures 3B, C**). Furthermore, correlation analysis indicated that expression of CD146 mRNA was significantly negatively correlated with its methylation status (**Figure 3D**). Among 18 predicted CpG sites of CD146, 15 CpG sites, including cg08187057, cg09042577, cg25484790, cg081861493, cg21096399, cg18890215, cg24827784, cg18165196, cg14976391, cg17466841, and cg11287851, were significantly correlated with the prognosis of ccRCC (**Table 4**). Consistently, CpG sites of CD146, including cg08187057, cg09042577, cg25484790, cg18890215, cg24827784, cg14976391, and cg17466841, showed higher methylation levels in ccRCC, indicating that the CD146 methylation in these CpG sites was correlated with poor prognosis in ccRCC patients (**Figure 3E**). These results revealed that the methylation levels of CD146 act as an effective prognostic biomarker for ccRCC, demonstrating that CD146 may have a pivotal role in tumor progression.

**Table 4:** The significant prognostic values of CpG in CD146.

Gene symbol	CpG Name	Hazard ratio	CI	LR test P value	UCSC Ref Gene Group	Relation to UCSC CpG Island
CD146	cg08187057	2.124	(1.444; 3.123)	1.6 e-04	3′UTR	N_Shore
	cg09042577	3.364	(1.944; 5.821)	7.6e-07	Body	N_Shore
	cg18165196	1.487	(0.913; 2.421)	0.097	Body	N_Shore
	cg25484790	2.848	(1.711; 4.740)	8.1e-06	Body	N_Shore
	cg08861493	1.959	(1.150; 3.337)	0.008	TSS1500	S_Shore
	cg21090399	1.794	(1.096; 2.905)	0.014	TSS1500	S_Shore
	cg18890215	1.898	(1.127; 3.196)	0.009	Body	S_Shore
	cg24827784	1.933	(1.224; 3.053)	0.003	Body	S_Shore
	cg03365354	1.236	(0.815; 1.874)	0.330	Body	Island
	cg03545206	0.437	(0.259; 0.738)	0.001	Body	Island
	cg04890495	0.658	(0.437; 0.989)	0.040	Body	Island
	cg14976391	1.895	(1.249; 2.873)	0.002	Body	Island
	cg17466841	2.482	(1.360; 4.530)	9e-04	Body	Island
	cg04470256	0.501	(0.340; 0.739)	4.6e-04	TSS200	Island
	cg02673010	0.329	(0.197; 0.549)	2.2e-06	TSS200	Island
	cg17622922	1.660	(1.131; 2.436)	0.096	TSS200	Island
	cg26864130	0.390	(0.225; 0.676)	2e-04	TSS200	Island
	cg11287851	1.824	(1.240; 2.682)	0.002	1stExon	Island

### CD146 Co-Expression Network in ccRCC

**Figure 4:**
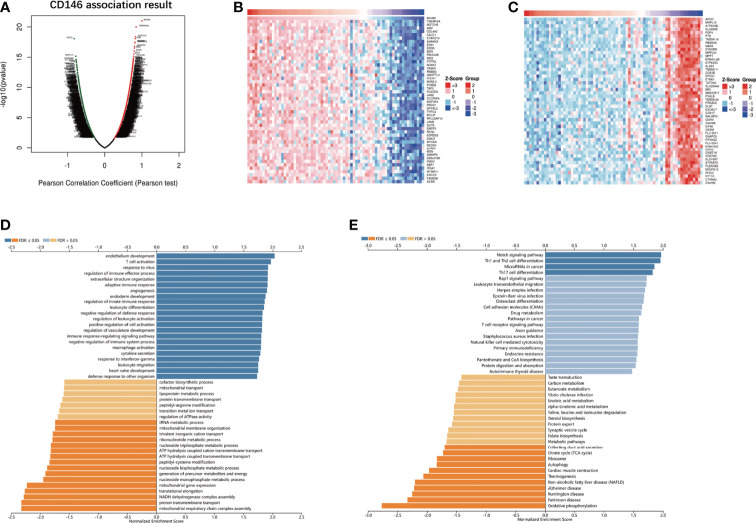
CD146 co-expressed genes and functional enrichment analysis. **(A)** Volcano map of co-expressed profiling of CD146 in ccRCC by the LinkedOmics database. **(B, C)** Heat map of top 50 positively **(B)** and 50 negatively **(C)** correlated genes with CD146 are displayed. **(D, E)** CD146 co-expression genes were annotated by Gene Ontology (GO) analysis **(D)** and Kyoto Encyclopedia of Genes and Genomes (KEGG) pathway analysis **(E)** available at LinkedOmics.

The results of the co-expression pattern of CD146 showed that 1904 genes were positively correlated with CD146, while 1696 genes were negatively correlated with CD146 (**Figure 4A**). Heat maps displayed the top 50 genes positively and negatively associated with CD146 (**Figures 4B, C**). GO term annotation showed that co-expressed genes of CD146 join mainly in endothelium development, response to virus, T cell activation, adaptive immune response, extracellular structure organization, angiogenesis, regulation of immune effector process, negative regulation of defense response, regulation of innate immune response, leukocyte differentiation, etc. (**Figure 4D**). KEGG pathway analysis indicated enrichment in the Notch signaling pathway, Th1 and Th2 cell differentiation, microRNAs in cancer, Th17 cell differentiation, leukocyte transendothelial migration, and the Rap1 signaling pathway (**Figure 4E**). These results indicate that the CD146 expression network influences the immune microenvironment greatly in ccRCC.

### CD146 Is Correlated With Immune Infiltration in ccRCC

**Figure 5:**
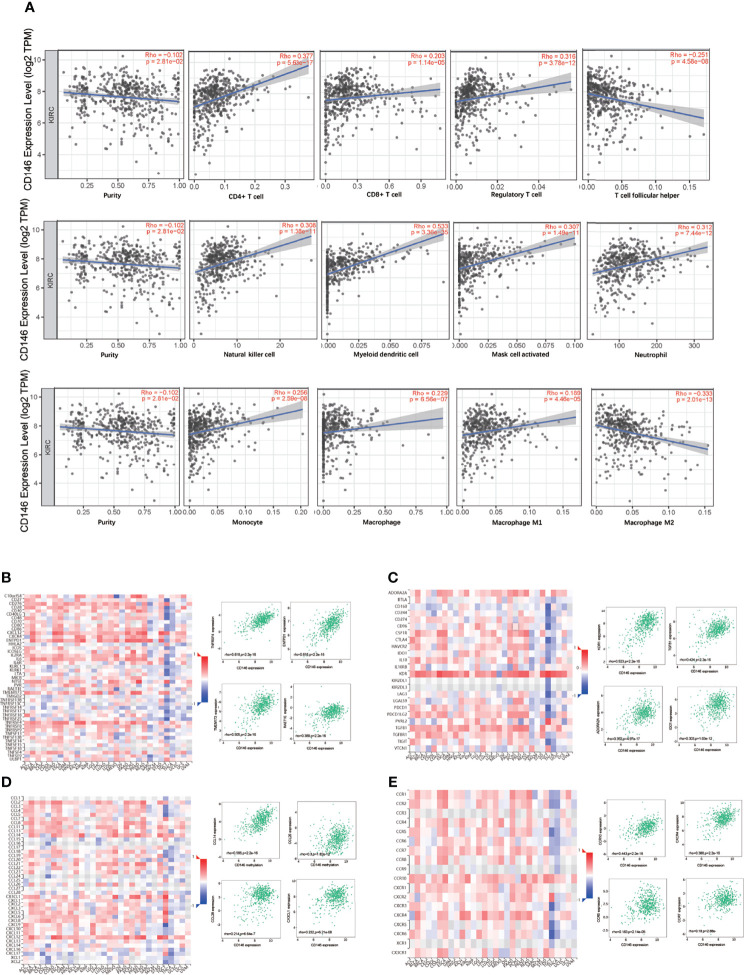
Correlation between CD146 with immune infiltration in ccRCC. **(A)** Correlation between CD146 expression and the abundance of tumor infiltrating immune cells in ccRCC available from the TIMER2.0 database. **(B, C)** Correlation between CD146 expression and immunostimulators **(B)** and immunoinhibitors **(C)** in ccRCC available from the TISIDB database. **(D, E)** Correlation between CD146 expression and chemokines **(D)** and chemokine receptors **(E)** in ccRCC available from the TISIDB database.

CD146 was positively correlated with infiltrating levels of neutrophils (rho = 0.312, p = 7.44e-12), myeloid dendritic cells (rho = 0.533, p = 3.36e-35), active CD4+ T cells (rho = 0.377, p = 5.63e-17), CD8+ T cells (rho = 0.203, p = 1.14e-05), Treg cells (rho = 0.316, p = 3.78e-12), Tfh cells (rho = -0.251, p = 4.58e-08), NK cells (rho = 0.308, p = 1.38e-11), activated mast cells (rho = 0.307, p = 1.49e-11), monocytes (rho = 0.256, p = 2.59e-08), macrophages (rho = 0.229, p = 6.56e-07), M1 macrophages (rho = 0.189, p = 4.46e-05), and M2 macrophages (rho = -0.333, p = 2.01e-13) (**Figure 5A**). In addition, CD146 was significantly correlated with the gene markers of monocytes, macrophages, M1 macrophages, M2 macrophages, neutrophils, NK cells, dendritic cells, Th1 cells, Th2 cells, Tfh cells, and Treg cells (**Table 5**). CD146 was significantly correlated with immune stimulators, such as TNFRSF4 (rho = 0.618, p < 2.2e-16), ENTPD1 (rho = 0.616, p < 2.2e-16), TMEM173 (rho = 0.505, p < 2.2e-16), and RAET1E (rho = 0.389, p < 2.2e-16) (**Figure 5B**). The expression of CD146 was also associated with immune inhibitors, including KDR (rho = 0.523, p < 2.2e-16), TGFB1 (rho = 0.424, p = 2.2e-16), ADORA2A (rho = 0.352, p < 4.97e-17), and IDO1 (rho = 0.303, p < 1.03e-12) (**Figure 5C**). CD146 expression was significantly correlated with CCL14 (rho = 0.566, p < 2.2e-16), CCL26 (rho = 0.300, p < 1.82e-12), CCL28 (rho = 0.214, p < 6.64e-7), and CX3CL1 (rho = 0.232, p < 6.21e-8) (**Figure 5D**). Meanwhile, CD146 expression was significantly associated with chemokine receptors, including CCR10 (rho = 0.443, p < 2.2e-16), CXCR4 (rho = 0.388, p < 2.2e-16), CCR6 (rho = 0.183, p < 2.14e-5), and CCR7 (rho = 0.180, p < 2.88e-5) (**Figure 5E**). These results support the findings that CD146 may function as an immunoregulatory factor in ccRCC.

**Table 5:** The correlations between CD146 and gene markers of immune cells in ccRCC and normal cells by GEPIA.

Description	Gene markers	None		Purity	
		Cor	*p*-Value	Cor	*p*-Value
CD8+T cell	CD8A	-0.005	0.910	-0.033	0.475
	CD8B	-0.028	0.521	-0.058	0.217
T cell (general)	CD3D	-0.001	0.973	-0.053	0.260
	CD3E	0.053	0.221	0.011	0.810
	CD2	0.024	0.584	-0.024	0.611
B cell	CD19	0.022	0.618	-0.006	0.904
	CD79A	0.026	0.542	-0.011	0.806
Monocyte	CD86	0.016	0.717	-0.034	0.466
	CD115(CSF1R)	0.165	***	0.124	**
TAM	CCL2	0.125	**	0.122	**
	CD68	-0.020	0.641	-0.052	0.267
	IL10	0.083	0.055	0.054	0.243
M1 macrophage	INOS(NOS2)	0.554	***	0.537	***
	IRF5	-0.214	***	-0.266	***
	COX2(PTGS2)	0.227	***	0.245	***
M2 macrophage	CD163	0.191	***	0.156	***
	VSIG4	0.079	0.069	0.024	0.610
	MS4A4A	0.154	***	0.120	*
Neutrophils	CD66b(CEACAM8)	0.087	*	0.095	0.041
	CD11b(ITGAM)	0.093	*	0.055	0.240
	CCR7	0.239	***	0.195	***
NK	KIR2DL1	0.316	***	0.305	***
	KIR2DL3	0.230	***	0.214	***
	KIR2DL4	0.061	0.161	0.045	0.339
	KIR3DL1	0.309	***	0.309	***
	KIR3DL2	0.275	***	0.254	***
	KIR3DL3	0.130	**	0.109	*
	KIR2DS4	0.274	***	0.265	***
Dendritic cell	HLA-DPB1	0.080	0.066	0.032	0.499
	HLA-DQB1	0.120	**	0.087	0.062
	HLA-DRA	0.048	0.264	0.001	0.984
	HLA-DPA1	0.094	*	0.048	0.302
	BDCA-1(CD1C)	0.245	***	0.214	***
	BDCA-4(NRP1)	0.683	***	0.668	***
	CD11c	-0.032	0.455	-0.053	0.259
Th1	T-bet (TBX21)	0.338	***	0.330	***
	STAT4	0.224	***	0.181	***
	STAT1	0.027	0.533	-0.013	0.779
	IFN-γ(IFNG)	-0.066	0.128	-0.109	*
	TNF-α(TNF)	0.032	0.454	-0.003	0.950
Th2	GATA3	0.043	0.326	0.053	0.259
	STAT6	0.228	***	0.226	*
	STAT5A	0.061	0.157	0.009	0.848
	IL13	0.101	*	0.125	**
Tfh	BCL6	0.303	***	0.306	***
	IL21	0.005	0.901	0.000	1.000
Th17	STAT3	0.437	***	0.437	***
	IL17A	0.024	0.577	0.003	0.942
Treg	FOXP3	0.004	0.919	-0.042	0.369
	CCR8	0.040	0.363	0.007	0.879
	STAT5B	0.452	***	0.458	***
	TGFβ(TGFB1)	0.506	***	0.504	***
T cell exhaustion	PD-1(PDCD1)	-0.076	0.078	-0.110	*
	CTLA4	-0.075	0.083	-0.124	**
	LAG3	-0.094	*	-0.133	**
	TIM3(HAVCR2)	0.024	0.578	-0.002	0.965
	GZMB	0.213	***	0.209	***

*p < 0.05. **p < 0.01. ***p < 0.001.

### CD146 Methylation Is Associated With Immunosuppressive Status in ccRCC

**Figure 6:**
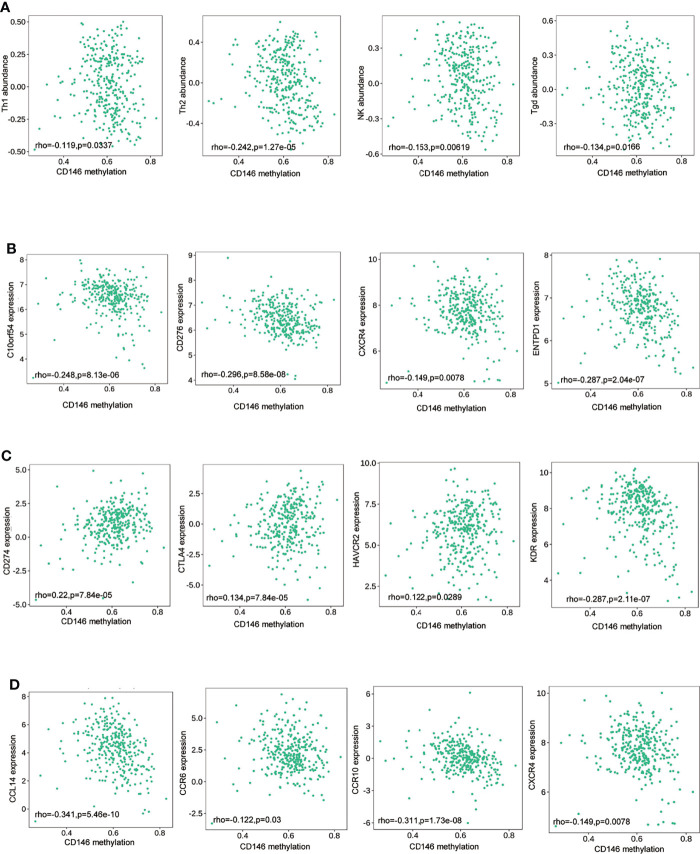
Association between the methylation status of CD146 with immune infiltrates in ccRCC. **(A)** Correlation of the methylation status of CD146 with NK cells, Type 1 T helper cells, Type 2 T helper cells, and γδT helper cells in ccRCC available from the TISIDB database. **(B–D)** Correlation of the methylation status of CD146 with immunostimulators **(B)** and immunoinhibitors **(C)** and chemokines/receptors **(D)** in ccRCC available from the TISIDB database.

As presented in the previous results, CD146 methylation in ccRCC correlates with prognosis in ccRCC. To elucidate the effect of CD146 methylation on the progression of ccRCC, we assessed the correlation of CD146 methylation with immune infiltration using TISIDB platforms. The result revealed that the methylation status of CD146 was negatively correlated with NK cells (rho = -0.153, p = 0.006), Th1 cells (rho = -0.119, p = 0.034), Th2 cells (rho = -0.242, p = 1.27e-5), and γδT cells (rho = -0.134, p = 0.0166) (**Figure 6A**). Similarly, the methylation status of CD146 was negatively associated with immune stimulators, such as C10orf54 (rho = -0.248, p = 8.13e-06), CD276 (rho = -0.296, p = 8.58e-08), CXCR4 (rho = -0.149, p = 0.0078), and ENTPD1 (rho = -0.287, p =2.04e-07) (**Figure 6B**), while being positively associated with immune inhibitors, such as CD274 (rho = 0.220, p = 7.84e-05), CTLA4 (rho = 0.134, p = 7.84e-05), HAVCR2 (rho = 0.122, p = 0.0289), and KDR (rho = 0.287, p = 2.11e-07) (**Figure 6C**). The methylation status of CD146 was also negatively associated with chemokines and receptors, such as CCL14 (rho = 0.341, p = 5.46e-10), CCR6 (rho = -0.122, p = 0.03), CCR10 (rho = -0.311, p = 1.73e-08), and CXCR4 (rho = -0.149, p = 0.0078) (**Figure 6D**). These results indicated that the methylation status of CD146 is positively associated with immunosuppressive status in ccRCC.

### CD146 Expression and Drug Response in Renal Cancer Cell Lines

**Figure 7:**
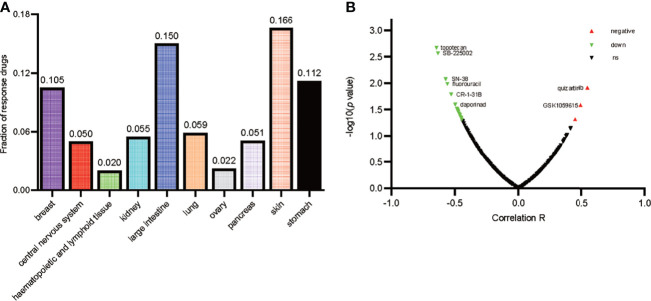
Drug response analysis of CD146. **(A)** The ratio of drugs correlated with CD146 expression in 10 various cancer cell line types including 30 cell lines is shown by histogram. **(B)** The correlation between CD146 expression and drug response in renal cancer cell lines is shown by volcano plot.

As is shown in **Figure 7A**, the ratio of drugs significantly correlated with CD146 expression in 10 different cancer cell line types is presented. The percentage of drugs significantly related with CD146 in renal cancer cell lines accounted for 5.5%. As is shown in **Figure 7B**, the relationship between CD146 and 545 drug response AUCs in renal cancer cell lines is presented. The drugs associated with CD146 in renal cancer cell lines are shown in **Table 6**. High CD146 expression was significantly correlated with a better response of inhibitors of topoisomerase I+II, including topotecan, SN-38, and etoposide. In addition, brivanib, inhibitor of VEGFR1/2, was also confirmed to be related to CD146 expression. Besides, inhibitors of DNA replication, including gemcitabine and clofarabine, were correlated with CD146 expression. Moreover, high CD146 expression was related to drug resistance of quizartinib, GSK1059615, BRD-K92856060, and AC55649. Overall, CD146 has the potential to become a therapeutic target for clinical treatment of ccRCC.

**Table 6:** Drug response related to CD146 expression in renal cancer cell lines.

Gene symbol	Compound	p-value	Correlation	Compound status	Target or activity of compound
CD146	gemcitabine	<0.000	-0.071	approved	inhibitor of DNA replication; inhibitor of ribonucleotide reductase, thymidylate synthetase, and cytidine monophosphate (UMP-CMP) kinase
	ML162	0.001	-0.703	probe	selectively kills engineered cells expressing mutant HRAS
	topotecan	0.002	-0.645	approved	inhibitor of topoisomerase I
	SB-225002	0.003	-0.633	probe	inhibitor of chemokine receptor 2
	SN-38	0.008	-0.572	probe	metabolite of irinotecan; inhibitor of topoisomerase I
	fluorouracil	0.010	-0.559	approved	pyrimidine analog; inhibitor of thymidylate synthase
	CR-1-31B	0.016	-0.530	probe	silvestrol analog; inhibits translation by modulating the eIF4F complex
	daporinad	0.025	-0.498	experimental	inhibitor of nicotinamide phosphoribosyltransferase
	GSK525762A	0.030	-0.485	probe	inhibitor of bromodomain (BRD) and extra-C terminal domain (BET) proteins
	BRD4132	0.031	-0.482	probe	screening hit
	clofarabine	0.032	-0.480	approved	inducer of DNA damage
	SNX-2112	0.033	-0.478	probe	inhibitor of HSP90alpha and HSP90beta
	WP1130	0.035	-0.474	probe	inhibitor of the deubiquitinase activity of USP9X, USP5, USP14, and UCH37
	I-BET151	0.035	-0.474	probe	inhibitor of bromodomain (BRD) and extra-C terminal domain (BET) proteins
	brivanib	0.038	-0.467	experimental	inhibitor of VEGFR 1/2
	serdemetan	0.040	-0.462	experimental	inhibitor of MDM2
	obatoclax	0.043	-0.456	experimental	inhibitor of MCL1, BCL2, and BCL-xL
	etoposide	0.046	-0.451	approved	inhibitor of topoisomerase II
	BIBR-1532	0.049	-0.446	probe	inhibitor of telomerase reverse transcriptase
	quizartinib	0.012	0.548	experimental	inhibitor of VEGFR3
	GSK1059615	0.027	0.495	experimental	inhibitor of PI3K and mTOR kinase activity
	BRD-K92856060	0.027	0.494	probe	screening hit
	AC55649	0.048	0.447	probe	agonist of retinoic acid receptor beta

## Discussion

Although surgical resection is the first-line therapy for clinically localized RCC, mortality after surgical treatment for RCC cannot be ignored. Elder patients and patients at high stage tend to have a higher 30-day mortality risk after surgery (47). Recent advances in understanding the molecular background of ccRCC have led to unprecedented progress in the diagnosis, prognosis, and therapy of ccRCC (48). Immunotherapies targeting the PD-L1/PD1 pathway have shown benefits in advanced ccRCC patients (49). However, patients that are on immunotherapy will eventually develop treatment resistance due to the immune evasion mechanism (50). In addition to the PD-L1/PD1 pathway, ample evidence supports the fact that many other molecules, such as siglec-15 and FGL1, also contribute to dysfunctional immunity in the TME (51, 52). Therefore, exploring the potential immune-related factors responsible for tumor immune escape may help to improve the prognosis of ccRCC patients. CD146, originally identified as a cell adhesion molecule, is widely involved in immune response, cell migration, and angiogenesis (15). Recent evidence also indicates that CD146 is overexpressed in malignant tumors and is associated with tumor progression (18, 53, 54). While the role of CD146 in ccRCC is unclear, we aimed at exploring the clinical significance and biological functions of CD146 in ccRCC by employing open-access databases for a comprehensive analysis.

According to the analysis of TCGA data and our IHC analysis, ccRCC showed a remarkable high expression of CD146 as mRNA and protein. The mechanism of CD146 upregulation in ccRCC is unclear. Luo Y. et al. reported that CD146 expression and the HIF-1α transcriptional program reinforce each other to physiologically enable pulmonary artery smooth muscle cells to adopt a more synthetic phenotype (11). As ccRCC is well elucidated for its VHL/HIF dysregulation and downstream signal abnormalities (55), we may speculate that the VHL/HIF pathway is the key upstream regulator of CD146 in ccRCC, which needs further verification. Researchers demonstrated that aberrant CpG island methylation of the CD146 gene promoter in breast cancer cells lines is involved in the expression control of CD146 (56). So, we investigated the promoter methylation level of CD146 in ccRCC using the UALCAN database. We found that the DNA methylation levels of CD146 in cancer tissues were significantly lower than that in normal samples, indicating that a low level of promoter methylation status of CD146 is responsible for the overexpression of CD146 in ccRCC. CD146 was reported to promote tumor progression, and elevated expression of CD146 predicted poor prognosis in cancer patients. To determine whether CD146 could be used as a prognostic marker in ccRCC, we investigated the prognosis of ccRCC patients with different CD146 expression levels. In our cohort. we observed that high CD146 expression in ccRCC tissues was associated with a poor prognosis in ccRCC patients. Multivariate Cox regression further confirmed that high CD146 expression is an independent adverse prognosis factor for ccRCC patients, suggesting that CD146 is a novel prognostic biomarker in ccRCC.

The genes in the same clique tend to be co-expressed and synergistically co-regulated. To unravel the biological functions of CD146, co-expression analysis and functional enrichment analysis were performed. Importantly, we identified genes involved in various immune-related processes, including T cell activation, adaptive immune response, regulation of immune effector process, regulation of innate immune response, regulation of leukocyte activation, macrophage activation, cytokine secretion, Th17 cell differentiation, Th1 and Th2 cell differentiation, and leukocyte transendothelial migration. These results indicate that CD146 may have complex regulatory roles in immune-related processes. To elucidate the role of CD146 in the TME, the relationships between CD146 and immune infiltration in ccRCC were analyzed by TIMER and TISIDB databases. Our results demonstrated that CD146 expression is not only significantly positively correlated with immune infiltration of the immune cell populations including neutrophil cells, monocytes, CD4+ T cells, CD8+ T cells, macrophages, myeloid dendritic cells, and NK cells, but also positively correlated with immunoinhibitors, such as KDR1, TGFB1, IDO1, and ADORA2A. Correlations between CD146 expression and gene markers of immune cells further revealed that CD146 has interactions with M2 macrophage cells and various functional T cells, such as Treg cells and exhausted T cells. These findings indicate that CD146 has dual inflammatory functions in ccRCC. High CD146 expression could enhance anti-tumor immunity by recruiting CD8+ T cells and NK cells to the TME, meanwhile, it induces exhausted phenotype T cells, Treg cells, and M2 type macrophages to accumulate in the TME, causing the inefficiency of anti-tumor immunity. Recent studies provided some insights that may explain the dual role of CD146 in ccRCC. Although CD146 could recruit neutrophils, macrophages, or activated T cells to the inflammatory microenvironment and exert a proinflammatory function (25, 57), the dynamic interaction between tumor cells and the TME could induce a chronic inflammation milieu that drives cancer development and progression (58, 59). The accumulation of Treg cells in tumors could inhibit anti-tumor immune responses. Our results showed that CD146 may be an important inducer of canonical features of T cell exhaustion. We observed that CD146 is positively correlated with key genes of exhausted T cells, including PD-1, LAG-3, TIM3, and GZMB. These genes are important immune checkpoint and immunotherapy targets in cancer therapy. Therefore, CD146 plays critical but different roles on the regulation of the TME, which is needed for identification at specific stages.

Dysregulation of DNA methylation of the epigenome will affect tumor immunogenicity and immune cells in the TME (60). Our study revealed that high methylation status of CD146 was often more frequently in high-grade and late-stage ccRCC, which may suggest that the pattern of methylation changes of CD146 promotes ccRCC progression. A previous study reported that aberrant methylation of the CD146 gene could potently induce the process of epithelial mesenchymal transition in cancer cells, thus contributing to tumor progression (56). To unravel the mechanism of CD146 methylation in promoting ccRCC progression, we analyzed the relationship between the methylation status of CD146 and immune infiltration. Our data showed that the methylation status of CD146 is negatively correlated with immune cells and immunostimulatory factors, while positively correlated with the immunoinhibitors. The methylation of CD146 may contribute to an immunosuppressive TME and promote tumor progression in ccRCC, which help to explain the high methylation status in late-stage and high-grade ccRCC tumors. The methylation of CD146 may be used as indicators of cancer immune infiltration and potential predictors of ccRCC patient response to immunotherapeutic drugs. In addition, we also found that CD146 methylation at certain CpG sites was correlated with poor prognosis in ccRCC patients, indicating that methylation levels of CD146 act as an effective prognostic biomarker for ccRCC.

Based on clinical and pathophysiological data, CD146 is a promising therapeutic target in ccRCC. The results of drug sensitivity analysis demonstrated that high CD146 expression in renal cancer cell lines was significantly correlated with a better response to brivanib, an inhibitor of VEGFR1/2. Previous studies showed that CD146, as a coreceptor of VEGFR2, participates in the angiogenesis of cancer *via* VEGF-induced VEGFR-2 phosphorylation (61, 62). Anti-CD146 and anti-VEGF therapy have a cumulative inhibitory effect on tumor angiogenesis, which may be new therapeutic models in ccRCC treatment. Besides, high CD146 expression was significantly correlated with a better response to inhibitors of topoisomerase, such as topotecan and SN-38. We speculate the expression of CD146 in ccRCC may be a new marker for increased sensitivity to topoisomerase inhibitors, which needs further validation. Importantly, we demonstrated a significant correlation of CD146 and drug resistance. For example, CD146 was significantly correlated with the resistance of GSK1059615. GSK1059615, an inhibitor of PI3K kinase activity, inhibited cancer cell growth, survival, proliferation, and cell cycle progression (63). The reason of the ineffectiveness of GSK1059615 in CD146 high expressing renal cancer cells may be that CD146 mediates mTORC2 activation, with no intervention of the PI3K and mTORC1 pathways, and promotes cell proliferation and survival (64). These results showed that CD146 may be a new therapeutic target for treating ccRCC patients.

In conclusion, this mining study revealed that CD146 is a prognosis-related biomarker for ccRCC. CD146 expression and methylation status of CD146 not only correlates with immune cell infiltration, but also correlates with immunomodulators and chemokines. Our study has certain limitations as follows: Firstly, gene expression analysis in our study, based on open-source databases, might not be sufficiently accurate. This calls for further experiments using *in vitro* and vivo models to explore the potential biological mechanisms of CD146 as well as tumor-immune interactions in ccRCC. Secondly, the role of CD146 in determining the clinical outcome to immunotherapy is still in need of further investigation. Thirdly, the scenario of methylation changes in the CD146 gene during development of ccRCC progression needs further study, as we cannot exclude the fact that CD146 acts as a tumor suppressor at the initial stage of carcinogenesis, as suggested by the study of Shih and others (65), and turns into an oncogene in the advanced stage. Therefore, our study highlights the novel immunomodulation function of CD146 in ccRCC.

## Data Availability Statement

The datasets presented in this study can be found in online repositories. The names of the repository/repositories and accession number(s) can be found in the article/supplementary material.

## Ethics Statement

Written informed consent was obtained from the individual(s) for the publication of any potentially identifiable images or data included in this article.

## Author Contributions

XZ and ZL designed the study. HY-F performed the experiment and data analysis. WT and HY-F performed clinical data analysis. ZL conducted the IHC and western blot assay. ZL and XZ contributed to manuscript writing, reviewing, and revision. HZ-L and XZ supervised the study. All authors contributed to the article and approved the submitted version.

## Funding

This study was financially supported by the National Natural Science Foundation of China (grant nos. 81972389 and 81770790).

## Conflict of Interest

The authors declare that the research was conducted in the absence of any commercial or financial relationships that could be construed as a potential conflict of interest.

## Publisher’s Note

All claims expressed in this article are solely those of the authors and do not necessarily represent those of their affiliated organizations, or those of the publisher, the editors and the reviewers. Any product that may be evaluated in this article, or claim that may be made by its manufacturer, is not guaranteed or endorsed by the publisher.

## References

[1] Siegel RL Miller KD Jemal A. Cancer Statistics, 2019. CA Cancer J Clin (2019) 69:7–34. doi: 10.3322/caac.21551 30620402

[2] Choueiri TK Halabi S Sanford BL Hahn O Michaelson MD Walsh MK. Cabozantinib Versus Sunitinib As Initial Targeted Therapy for Patients With Metastatic Renal Cell Carcinoma of Poor or Intermediate Risk: The Alliance A031203 CABOSUN Trial. J Clin Oncol (2017) 35:591–7. doi: 10.1200/JCO.2016.70.7398 PMC5455807 28199818

[3] Motzer RJ Barrios CH Kim TM Falcon S Cosgriff T Harker WG. Phase II Randomized Trial Comparing Sequential First-Line Everolimus and Second-Line Sunitinib Versus First-Line Sunitinib and Second-Line Everolimus in Patients With Metastatic Renal Cell Carcinoma. J Clin Oncol (2014) 32:2765–72. doi: 10.1200/JCO.2013.54.6911 PMC5569681 25049330

[4] Yoshihara K Shahmoradgoli M Martínez E Vegesna R Kim H Torres-Garcia W. Inferring Tumour Purity and Stromal and Immune Cell Admixture From Expression Data. Nat Commun (2013) 4:2612. doi: 10.1038/ncomms3612 24113773 PMC3826632

[5] Motzer RJ Penkov K Haanen J Rini B Albiges L Campbell MT. Avelumab Plus Axitinib Versus Sunitinib for Advanced Renal-Cell Carcinoma. N Engl J Med (2019) 380:1103–15. doi: 10.1056/NEJMoa1816047 PMC6716603 30779531

[6] Rini BI Plimack ER Stus V Gafanov R Hawkins R Nosov D. Pembrolizumab Plus Axitinib Versus Sunitinib for Advanced Renal-Cell Carcinoma. N Engl J Med (2019) 380:1116–27. doi: 10.1056/NEJMoa1816714 30779529

[7] Turajlic S Xu H Litchfield K Rowan A Horswell S Chambers T. Deterministic Evolutionary Trajectories Influence Primary Tumor Growth: TRACERx Renal. Cell (2018) 173:595–610. doi: 10.1016/j.cell.2018.03.043 29656894 PMC5938372

[8] Mitchell TJ Turajlic S Rowan A Nicol D Farmery JHR O'Brien T. Timing the Landmark Events in the Evolution of Clear Cell Renal Cell Cancer: TRACERx Renal. Cell (2018) 173:611–623.e17. doi: 10.1016/j.cell.2018.02.020 29656891 PMC5927631

[9] Brahmer A Neuberger E Esch-Heisser L Haller N Jorgensen MM Baek R. Platelets, Endothelial Cells and Leukocytes Contribute to the Exercise-Triggered Release of Extracellular Vesicles Into the Circulation. J Extracell Vesicles (2019) 8:1615820. doi: 10.1080/20013078.2019.1615820 31191831 PMC6542154

[10] Zheng B Ohuchida K Chijiiwa Y Zhao M Mizuuchi Y Cui L. CD146 Attenuation in Cancer-Associated Fibroblasts Promotes Pancreatic Cancer Progression. Mol Carcinog (2016) 55:1560–72. doi: 10.1002/mc.22409 26373617

[11] Luo Y Teng X Zhang L Chen J Liu Z Chen X. CD146-HIF-1α Hypoxic Reprogramming Drives Vascular Remodeling and Pulmonary Arterial Hypertension. Nat Commun (2019) 10:3551. doi: 10.1038/s41467-019-11500-6 31391533 PMC6686016

[12] Dagur PK Tatlici G Gourley M Samsel L Raghavachari N Liu P. CD146+ T Lymphocytes Are Increased in Both the Peripheral Circulation and in the Synovial Effusions of Patients With Various Musculoskeletal Diseases and Display Pro-Inflammatory Gene Profiles. Cytometry B Clin Cytom (2010) 78:88–95. doi: 10.1002/cyto.b.20502 19834966 PMC2904479

[13] Luo Y Duan H Qian Y Feng L Wu Z Wang F. Macrophagic CD146 Promotes Foam Cell Formation and Retention During Atherosclerosis. Cell Res (2017) 27:352–72. doi: 10.1038/cr.2017.8 PMC5339843 28084332

[14] Sun Z Ji N Ma Q Zhu R Chen Z Wang Z. Epithelial-Mesenchymal Transition in Asthma Airway Remodeling Is Regulated by the IL-33/CD146 Axis. Front Immunol (2020) 11:1598. doi: 10.3389/fimmu.2020.01598 32793232 PMC7387705

[15] Wang Z Yan X. CD146, a Multi-Functional Molecule Beyond Adhesion. Cancer Lett (2013) 330:150–62. doi: 10.1016/j.canlet.2012.11.049 23266426

[16] Liu WF Ji SR Sun JJ Zhang Y Liu ZY Liang AB. CD146 Expression Correlates With Epithelial-Mesenchymal Transition Markers and a Poor Prognosis in Gastric Cancer. Int J Mol Sci (2012) 13:6399–406. doi: 10.3390/ijms13056399 PMC3382746 22754372

[17] Rapanotti MC Campione E Spallone G Orlandi A Bernardini S Bianchi L. Minimal Residual Disease in Melanoma: Circulating Melanoma Cells and Predictive Role of MCAM/MUC18/MelCAM/Cd146. Cell Death Discovery (2017) 3:17005. doi: 10.1038/cddiscovery.2017.5 28280601 PMC5337524

[18] Zhang F Wang J Wang X Wei N Liu H Zhang X. CD146-Mediated Acquisition of Stemness Phenotype Enhances Tumour Invasion and Metastasis After EGFR-TKI Resistance in Lung Cancer. Clin Respir J (2019) 13:23–33. doi: 10.1111/crj.12976 30480362

[19] Chen Y Sumardika IW Tomonobu N Kinoshita R Inoue Y Iioka H. Critical Role of the MCAM-ETV4 Axis Triggered by Extracellular S100A8/A9 in Breast Cancer Aggressiveness. Neoplasia (2019) 21:627–40. doi: 10.1016/j.neo.2019.04.006 PMC6520639 31100639

[20] Wang P Luo Y Duan H Xing S Zhang J Lu D. MicroRNA 329 Suppresses Angiogenesis by Targeting CD146. Mol Cell Biol (2013) 33:3689–99. doi: 10.1128/MCB.00343-13 PMC3753872 23878390

[21] Kaspi E Heim X Granel B Guillet B Stalin J Nollet M. Identification of CD146 as a Novel Molecular Actor Involved in Systemic Sclerosis. J Allergy Clin Immunol (2017) 140:1448–51.e6. doi: 10.1016/j.jaci.2017.04.046 28606586

[22] Neidhart M Wehrli R Brühlmann P Michel BA Gay RE Gay S. Gay Synovial Fluid CD146 (MUC18), a Marker for Synovial Membrane Angiogenesis in Rheumatoid Arthritis. Arthritis Rheum (1999) 42:622–30. doi: 10.1002/1529-0131(199904)42:4<622:AID-ANR4>3.0.CO;2-Y 10211875

[23] Tsiolakidou G Koutroubakis IE Tzardi M Kouroumalis EA. Increased Expression of VEGF and CD146 in Patients With Inflammatory Bowel Disease. Dig Liver Dis (2008) 40:673–9. doi: 10.1016/j.dld.2008.02.010 18374637

[24] Bardin N Blot-Chabaud M Despoix N Kebir A Harhouri K Arsanto JP. CD146 and its Soluble Form Regulate Monocyte Transendothelial Migration. Arterioscler Thromb Vasc Biol (2009) 29:746–53. doi: 10.1161/ATVBAHA.108.183251 19229070

[25] Guezguez B Vigneron P Lamerant N Kieda C Jaffredo T Dunon D. Dual Role of Melanoma Cell Adhesion Molecule (MCAM)/CD146 in Lymphocyte Endothelium Interaction: MCAM/CD146 Promotes Rolling *via* Microvilli Induction in Lymphocyte and Is an Endothelial Adhesion Receptor. J Immunol (2007) 179:6673–85. doi: 10.4049/jimmunol.179.10.6673 17982057

[26] Murata M. Inflammation and Cancer. Environ Health Prev Med (2018) 23:50. doi: 10.1186/s12199-018-0740-1 30340457 PMC6195709

[27] Netti GS Lucarelli G Spadaccino F Castellano G Gigante M Divella C. PTX3 Modulates the Immunoflogosis in Tumor Microenvironment and Is a Prognostic Factor for Patients With Clear Cell Renal Cell Carcinoma. Aging (2020) 12:7585–602. doi: 10.18632/aging.103169 PMC7202504 32345771

[28] Falagario UG Busetto GM Netti GS Sanguedolce F Selvaggio O Infante B. Prospective Validation of Pentraxin-3 as a Novel Serum Biomarker to Predict the Risk of Prostate Cancer in Patients Scheduled for Prostate Biopsy. Cancers (2021) 13:1611–20. doi: 10.3390/cancers13071611 PMC8036446 33807333

[29] Lee A Lee HJ Huang HH Tay KJ Lee LS Sim SPA. Prognostic Significance of Inflammation-Associated Blood Cell Markers in Nonmetastatic Clear Cell Renal Cell Carcinoma. Clin Genitourin Cancer (2020) 18:304–13. doi: 10.1016/j.clgc.2019.11.013 31892491

[30] Krishna Y Acha-Sagredo A Sabat-Pośpiech D Kipling N Clarke K Figueiredo CR. Transcriptome Profiling Reveals New Insights Into the Immune Microenvironment and Upregulation of Novel Biomarkers in Metastatic Uveal Melanoma. Cancers (2020) 12:2832–54. doi: 10.3390/cancers12102832 PMC7650807 33008022

[31] Li T Fan J Wang B Traugh N Chen Q Liu JS. TIMER: A Web Server for Comprehensive Analysis of Tumor-Infiltrating Immune Cells. Cancer Res (2017) 77:e108–10. doi: 10.1158/0008-5472.Can-17-0307 PMC6042652 29092952

[32] Sousa S Määttä J. The Role of Tumour-Associated Macrophages in Bone Metastasis. J Bone Oncol (2016) 5:135–8. doi: 10.1016/j.jbo.2016.03.004 PMC5063225 27761375

[33] Danaher P Warren S Dennis L D'Amico L White A Disis ML. Gene Expression Markers of Tumor Infiltrating Leukocytes. J Immunother Cancer (2017) 5:18. doi: 10.1186/s40425-017-0215-8 28239471 PMC5319024

[34] Siemers NO Holloway JL Chang H Chasalow SD Ross-MacDonald PB Voliva CF. Genome-Wide Association Analysis Identifies Genetic Correlates of Immune Infiltrates in Solid Tumors. PloS One (2017) 12:e0179726. doi: 10.1371/journal.pone.0179726 28749946 PMC5531551

[35] Győrffy B Bottai G Fleischer T Munkácsy G Budczies J Paladini L. Aberrant DNA Methylation Impacts Gene Expression and Prognosis in Breast Cancer Subtypes. Int J Cancer (2016) 138:87–97. doi: 10.1002/ijc.29684 26174627

[36] Paner GP Stadler WM Hansel DE Montironi R Lin DW Amin MB. Updates in the Eighth Edition of the Tumor-Node-Metastasis Staging Classification for Urologic Cancers. Eur Urol (2018) 73:560–9. doi: 10.1016/j.eururo.2017.12.018 29325693

[37] Fuhrman SA Lasky LC Limas C. Limas Prognostic Significance of Morphologic Parameters in Renal Cell Carcinoma. Am J Surg Pathol (1982) 6:655–63. doi: 10.1097/00000478-198210000-00007 7180965

[38] Zhao T Ren H Li J Chen J Zhang H Xin W. LASP1 Is a HIF1α Target Gene Critical for Metastasis of Pancreatic Cancer. Cancer Res (2015) 75:111–9. doi: 10.1158/0008-5472.CAN-14-2040 PMC4286473 25385028

[39] Gao Y Li H Ma X Fan Y Ni D Zhang Y. KLF6 Suppresses Metastasis of Clear Cell Renal Cell Carcinoma *via* Transcriptional Repression of E2F1. Cancer Res (2017) 77:330–42. doi: 10.1158/0008-5472.CAN-16-0348 27780824

[40] Huang QB Ma X Li HZ Ai Q Liu SW Zhang Y. Endothelial Delta-Like 4 (DLL4) Promotes Renal Cell Carcinoma Hematogenous Metastasis. Oncotarget (2014) 5:3066–75. doi: 10.18632/oncotarget.1827 PMC4102792 24931473

[41] Chandrashekar DS Bashel B Balasubramanya SAH Creighton CJ Ponce-Rodriguez I Chakravarthi BVSK. UALCAN: A Portal for Facilitating Tumor Subgroup Gene Expression and Survival Analyses. Neoplasia (2017) 19:649–58. doi: 10.1016/j.neo.2017.05.002 PMC5516091 28732212

[42] Klutstein M Nejman D Greenfield R Cedar H. DNA Methylation in Cancer and Aging. Cancer Res (2016) 76:3446–50. doi: 10.1158/0008-5472.CAN-15-3278 27256564

[43] Vasaikar SV Straub P Wang J Zhang B. LinkedOmics: Analyzing Multi-Omics Data Within and Across 32 Cancer Types. Nucleic Acids Res (2018) 46:D956–d963. doi: 10.1093/nar/gkx1090 29136207 PMC5753188

[44] Ru B Wong CN Tong Y Zhong JY Zhong SSW Wu WC. TISIDB: An Integrated Repository Portal for Tumor-Immune System Interactions. Bioinformatics (2019) 35:4200–2. doi: 10.1093/bioinformatics/btz210 30903160

[45] Mukaka MM. Statistics Corner: A Guide to Appropriate Use of Correlation Coefficient in Medical Research. Malawi Med J (2012) 24:69–71. 23638278 PMC3576830

[46] Rees MG Seashore-Ludlow B Cheah JH Adams DJ Price EV Gill S. Correlating Chemical Sensitivity and Basal Gene Expression Reveals Mechanism of Action. Nat Chem Biol (2016) 12:109–16. doi: 10.1038/nchembio.1986 PMC4718762 26656090

[47] Falagario UG Veccia A Cormio L Simeone C Carbonara U Crocerossa F. Nomogram Predicting 30-Day Mortality After Nephrectomy in the Contemporary Era: Results From the SEER Database. Int J Urol (2021) 28:309–14. doi: 10.1111/iju.14461 33319434

[48] Miao D Margolis CA Gao W Voss MH Li W Martini DJ. Genomic Correlates of Response to Immune Checkpoint Therapies in Clear Cell Renal Cell Carcinoma. Science (2018) 359:801–6. doi: 10.1126/science.aan5951 PMC6035749 29301960

[49] Dong H Strome SE Salomao DR Tamura H Hirano F Flies DB. Tumor-Associated B7-H1 Promotes T-Cell Apoptosis: A Potential Mechanism of Immune Evasion. Nat Med (2002) 8:793–800. doi: 10.1038/nm730 12091876

[50] Sharma P Hu-Lieskovan S Wargo JA Ribas A. Primary, Adaptive, and Acquired Resistance to Cancer Immunotherapy. Cell (2017) 168:707–23. doi: 10.1016/j.cell.2017.01.017 PMC5391692 28187290

[51] Wang J Sun J Liu LN Flies DB Nie X Toki M. Siglec-15 as an Immune Suppressor and Potential Target for Normalization Cancer Immunotherapy. Nat Med (2019) 25:656–66. doi: 10.1038/s41591-019-0374-x PMC7175920 30833750

[52] Wang J Sanmamed MF Datar I Su TT Ji L Sun J. Fibrinogen-Like Protein 1 Is a Major Immune Inhibitory Ligand of LAG-3. Cell (2019) 176:334–47.e12. doi: 10.1016/j.cell.2018.11.010 PMC6365968 30580966

[53] Zoni E Astrologo L Ng CKY Piscuoglio S Melsen J Grosjean J. Therapeutic Targeting of CD146/MCAM Reduces Bone Metastasis in Prostate Cancer. Mol Cancer Res (2019) 17:1049–62. doi: 10.1158/1541-7786.MCR-18-1220 30745464

[54] Chen J Dang Y Feng W Qiao C Liu D Zhang T. SOX18 Promotes Gastric Cancer Metastasis Through Transactivating MCAM and CCL7. Oncogene (2020) 39:5536–52. doi: 10.1038/s41388-020-1378-1 32616889

[55] Schödel J Grampp S Maher ER Moch H Ratcliffe PJ Russo P. Hypoxia, Hypoxia-Inducible Transcription Factors, and Renal Cancer. Eur Urol (2016) 69:646–57. doi: 10.1016/j.eururo.2015.08.007 PMC5012644 26298207

[56] Dudzik P Trojan SE Ostrowska B Lasota M Dulińska-Litewka J Laidler P. Aberrant Promoter Methylation may be Responsible for the Control of CD146 (MCAM) Gene Expression During Breast Cancer Progression. Acta Biochim Pol (2019) 66:619–25. doi: 10.18388/abp.2019-2907 31826047

[57] Stevenson C Jiang D Schaefer N Ito Y Berman R Sanchez A. MUC18 Regulates IL-13-Mediated Airway Inflammatory Response. Inflammation Res (2017) 66:691–700. doi: 10.1007/s00011-017-1050-6 PMC5612446 28451734

[58] Fridlender ZG Sun J Kim S Kapoor V Cheng G Ling L. Polarization of Tumor-Associated Neutrophil Phenotype by TGF-Beta: "N1" Versus "N2" TAN. Cancer Cell (2009) 16:183–94. doi: 10.1016/j.ccr.2009.06.017 PMC2754404 19732719

[59] Mehla K Singh PK. Singh Metabolic Regulation of Macrophage Polarization in Cancer. Trends Cancer (2019) 5:822–34. doi: 10.1016/j.trecan.2019.10.007 PMC7187927 31813459

[60] Hogg SJ Beavis PA Dawson MA Johnstone RW. Johnstone Targeting the Epigenetic Regulation of Antitumour Immunity. Nat Rev Drug Discovery (2020) 19:776–800. doi: 10.1038/s41573-020-0077-5 32929243

[61] Jiang T Zhuang J Duan H Luo Y Zeng Q Fan K. CD146 Is a Coreceptor for VEGFR-2 in Tumor Angiogenesis. Blood (2012) 120:2330–9. doi: 10.1182/blood-2012-01-406108 22718841

[62] Zeng Q Wu Z Duan H Jiang X Tu T Lu D. Impaired Tumor Angiogenesis and VEGF-Induced Pathway in Endothelial CD146 Knockout Mice. Protein Cell (2014) 5:445–56. doi: 10.1007/s13238-014-0047-y PMC4026419 24756564

[63] Bei S Li F Li H Li J Zhang X Sun Q. Inhibition of Gastric Cancer Cell Growth by a PI3K-mTOR Dual Inhibitor GSK1059615. Biochem Biophys Res Commun (2019) 511:13–20. doi: 10.1016/j.bbrc.2019.02.032 30765226

[64] Xu W Hua H Chiu YH Li G Zhi H Yu Z. CD146 Regulates Growth Factor-Induced Mtorc2 Activity Independent of the PI3K and Mtorc1 Pathways. Cell Rep (2019) 29:1311–22.e5. doi: 10.1016/j.celrep.2019.09.047 31665642

[65] Shih LM Hsu MY Palazzo JP Herlyn M. The Cell-Cell Adhesion Receptor Mel-CAM Acts as a Tumor Suppressor in Breast Carcinoma. Am J Pathol (1997) 151:745–51. PMC1857834 9284823

